# Therapeutic Effects of Saline Groundwater Solution Baths on Atopic Dermatitis: A Pilot Study

**DOI:** 10.1155/2020/8303716

**Published:** 2020-06-10

**Authors:** Jisook Yoo, Ji Young Choi, Bo Young Lee, Chang Ho Shin, Jung-Won Shin, Chang Hun Huh, Jung-Im Na

**Affiliations:** ^1^Department of Dermatology, Seoul National University Bundang Hospital, Seongnam, Gyeonggi 13520, Republic of Korea; ^2^Aribio Co. Ltd., Seongnam, Gyeonggi 13487, Republic of Korea

## Abstract

**Background:**

Saline groundwater, collected from the east coast of Korea, has been shown to have protective effects against 2,4-dinitrochlorobenzene- (DNCB-) induced atopic dermatitis-like skin lesions in the murine model.

**Objectives:**

To determine the effects of saline groundwater solution baths as a treatment of mild-to-moderate atopic dermatitis.

**Methods:**

Twenty-four subjects with mild-to-moderate atopic dermatitis were instructed to take a bath in saline groundwater solution for 20 minutes per day for two weeks. Evaluations were performed at baseline and week 2, including SCORing Atopic Dermatitis (SCORAD) index, corneometry, transepidermal water loss, visual analogue scale for pruritus, and collection of adverse events.

**Results:**

Subjects showed significant improvement with respect to the SCORAD index, skin hydration, transepidermal water loss, and pruritus at week 2 when compared with the baseline.

**Conclusion:**

Baths in saline groundwater solution may be an alternative therapeutic strategy for treating atopic dermatitis.

## 1. Introduction

Atopic dermatitis (AD) is a chronic inflammatory skin disease affecting a large portion of the population [1]. Due to decreased epidermal barrier function, patients with AD tend to have dry skin and are generally prone to skin infections, which in turn exacerbate the inflammation [2]. Baths not only remove impurities from the skin, but they can also decrease the risks of infection and provide skin hydration; therefore, they can be considered a vital treatment for AD [3].

The mainstays of treatment for AD include topical steroids, emollients, topical calcineurin inhibitors, systemic antihistamines, and systemic immunomodulators [4–6]. However, the effects of these treatments are limited, and long-term use has been associated with side effects [5]. Researchers have tried to find alternative treatments including bathing in Dead Seawater and spring water. These have been proven to be effective in ameliorating symptoms in patients with AD and other skin diseases [7, 8]. According Proksch et al. [8], patients who took baths in Dead Seawater for 15 minutes showed higher water contents in the epidermis, better epidermal barrier function, and decreased skin inflammation compared with the group who took baths in regular tap water. Portales et al. also reported that bathing in spring water from Avene decreased serum inflammatory cytokines [9]. Saline groundwater (SGW) refers to groundwater collected from the coastal aquifers. SGW is rich in minerals, such as Ca and Mg, and is lower in salinity and pH compared with seawater [10, 11]. According to a 2014 study conducted by the Korea Institute of Ocean Science and Technology, SGW from Uljin, a town on the eastern coast of South Korea, showed protective effects against 2,4-dinitrochlorobenzene- (DNCB-) induced AD-like skin lesions in the murine model [12]. The aim of this study is to examine the effects of SGW from Uljin in mild-to-moderate AD patients. To the best of our knowledge, this is the first study to evaluate the effects of SGW on AD patients.

## 2. Materials and Methods

### 2.1. Study Design and Subjects

This pilot study was designed as an uncontrolled before-after study. Volunteers aged between 4 and 40 years with mild to moderate AD participated [13]. Subjects with severe AD or history of treatment with systemic steroid or immunosuppressive agents within the past four weeks, or a history of allergic reaction to high mineral bath were excluded from the study. The study protocol was approved by the ethics committee of the Seoul National University Bundang Hospital (B-1807/478-005). Informed consent was obtained from all participants.

### 2.2. Saline Groundwater

The SGW used in this study is from Uljin, South Korea. SGW from Uljin contained various minerals, including calcium (1770.00 mg/L), magnesium (1010.00 mg/L), potassium (90.465 mg/L), and sodium (90.4 mg/L). After filtering, SGW was concentrated to 50 times for further use.

### 2.3. Saline Groundwater Solution Bath

**Figure 1:**
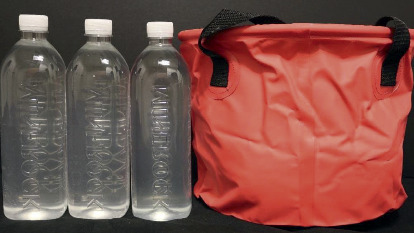
Fifty-fold concentrated SGW in 1 L bottles and a 20 L-sized bucket provided to the participants for dilution.

Participants were instructed to take a 20 minute bath in SGW solution once a day for two weeks. SGW solution was prepared by diluting 1L of 50-fold SGW in 100 L of warm tap water in the bath tub, resulting in half the original concentration. Participants were provided with a 20 L bucket to aid in making the dilutions (Figure 1). Participants were instructed to maintain their skincare routine during the trial, i.e., if they usually used cleansers, they were asked to keep using them before baths, and if they did not normally use them, they were asked to go straight into the bathtub. After the bath, they were instructed to gently wipe their body with a dry towel and apply moisturizers as usual. Introducing new topical agents was not allowed during the trial.

### 2.4. Measurement

Participants were scheduled to visit at baseline and at week 2. The following measurements were carried out at each visit: SCORing Atopic Dermatitis (SCORAD) index; visual analogue scale (VAS) for pruritus; corneometry (CorneometerVR CM825, Courage & Khazaka, Germany); and transepidermal water loss (TEWL, Tewameter, Courage and Khazaka, Germany). The SCORAD index is a standardized assessment tool for the severity of AD [14]. For evaluation of pruritus, participants were asked to rate their itchiness on a visual analogue scale (VAS). Corneometry measures the hydration level of stratum corneum, while TEWL assesses the stratum corneum barrier function. Both corneometry and TEWL were measured at the antecubital fossa of the left arm.

### 2.5. Statistical Analysis

All statistical analyses were performed using GraphPad Prism 8.1.0 (GraphPad Software, Inc., CA).

## 3. Results

**Figure 2:**
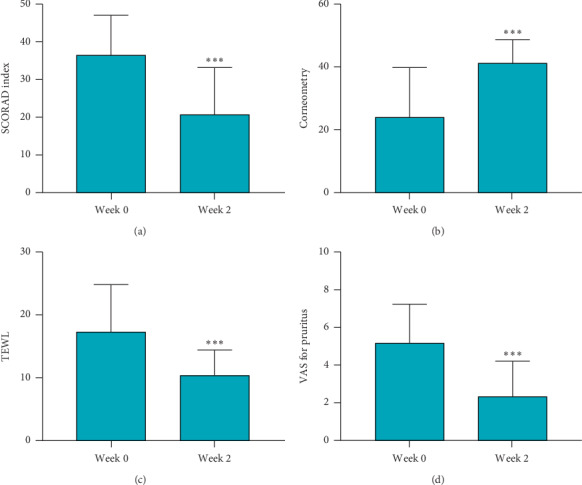
The mean SCORAD index score, corneometry, TEWL, and VAS for pruritus of atopic dermatitis patients treated with SGW bath. (a) SCORAD index score decreased after SGW bath treatment. The average SCORAD index score decreased from 36.6 ± 10.80 to 20.73 ± 12.41 at week 2. (b) Skin hydration increased from 24.72 ± 16.47 to 42.67 ± 7.66. (c) TEWL was reduced from 18.80 ± 8.21 to 11.35 ± 4.33 after treatment. (d) Pruritus improved after treatment. ^*∗∗∗*^*P* < 0.005 (SCORAD, SCORing of Atopic Dermatitis; TEWL, transepidermal water loss; SGW, saline groundwater).

A total of 24 participants were initially enrolled, of which, 23 completed the study. Participants' age ranged between 4 and 37, with an average of 13.2 years. The SCORAD index score significantly decreased from 36.3 ± 10.80 to 20.73 ± 12.41 after the treatment; there was a significant improvement in the severity of AD (*P* < 0.0001, Figure 2(a)). Skin hydration significantly increased compared with the baseline (24.72 ± 16.47 at baseline; 42.67 ± 7.66 at week 2, *P* < 0.0001, Figure 2(b)), and TEWL significantly decreased after treatment (18.80 ± 8.21 at baseline; 11.35 ± 4.33 at week 2, *P* = 0.0012, Figure 2(c)). The increase in skin hydration and decrease in TEWL suggest that the epidermal barrier function may have improved. Participants reported a significant decrease of pruritus. The average VAS for pruritus was 5.65 ± 2.29 at baseline, which was decreased to 2.57 ± 2.06 at week 2 (*P* < 0.0001, Figure 2(d)). As for safety, one patient dropped out due to exacerbation of existing lesions. No serious adverse events were reported throughout the study period.

## 4. Discussion

AD is a chronic inflammatory skin disease affecting a large portion of the population. Skin barrier defect is the hallmark of AD [15]. According to the “outside-inside-outside” model for the pathogenesis of AD [16], a skin barrier defect may cause inflammation, allowing allergens and microbials to penetrate into the skin, which further exacerbates both inflammation and the skin barrier defect. Therefore, it is important to consider both inflammation control and skin barrier function restoration when treating AD. Bathing is an important baseline treatment for AD. Various bathing additives have been studied for AD [17]; however, it is difficult to compare the effects of each bathing method because the study design and the evaluation method are not uniform. The use of dilute 0.005% bleach baths has shown efficacy in reducing AD severity; however, when comparing 4-week use of either the bleach bath versus water bath in patients with AD, there was no significant difference in AD severity [3].

According to Kim et al. [18], green tea baths in AD patients have reduced SCORAD from 47.05 ± 7.94 at baseline to 23.4 ± 3.83 at week 4 with reduction of 50.3%. However, only four patients were enrolled in the study and the results did not show statistical significance.

Thalassotherapy, a therapy using seawater, has been developed since the mid-19^th^ century [19]. Dead Seawater was shown to be effective in alleviating symptoms of psoriasis and AD, with or without solar irradiation [8, 20]. It improves skin barrier function through high concentrations of magnesium and calcium ions, influencing epidermal proliferation and differentiation [21, 22]. Magnesium ions in the SGW are also known to have anti-inflammatory effect, inhibiting contact dermatitis, and antigen-presenting ability to the Langerhans cells [23, 24]. An *in vivo* study conducted by Bak et al. showed that topical application of deep seawater significantly reduced T helper (Th) 2 cytokines, serum IgE levels, and clinical severity indexes of AD-like skin lesions in mice, showing anti-inflammatory effects of deep seawater [25]. Seawater, other than deep seawater, is also shown to alleviate AD in mouse models [12]. SGW has the following advantages over seawater. SGW has higher calcium concentration and lower pH than seawater, which together may provide greater benefit to the restoration of skin barrier function. Moreover, due to natural filtration through the sediment, it has lower microbial contamination than seawater, which makes it easier to prepare as a bath product [10, 11]. SGW baths are shown to have better therapeutic effects than seawater in a murine model of AD [12]. In the AD mouse model, bathing in SGW from Uljin demonstrated anti-inflammatory and antioxidative effects, in a concentration-dependent manner [11]. SGW decreased total serum IgE, T helper 2 cytokine levels, and clinical severity of eczema. It is also shown to restore cutaneous glutathione level and decrease superoxide anion in the skin, acting as an antioxidant [11]. SGW is also abundant and relatively cheap.

In our study, the use of SGW baths significantly reduced the severity and pruritus of AD. It also improved epidermal barrier function, which was confirmed by increased stratum corneum water content and reduced TEWL. Patients tolerated the SGW baths well. Although this is only an uncontrolled pilot study, it is meaningful that it demonstrated the potential of SGW bath as a safe and cheap alternative therapy for AD.

## 5. Conclusions

Here, we showed that baths using diluted SGW improved the clinical severity and symptoms of AD with statistical significance. The water content in the epidermis increased, while TEWL decreased, indicating improved epidermal barrier function. Participants tolerated SGW baths well without any side effects. This result shows the potential of SGW baths as a safe and cheap alternative therapy for AD.

## Data Availability

The data used to support the findings of this study are available from the corresponding author upon request.

## References

[1] Li C., Lasse S., Lee P. (2010). Development of atopic dermatitis-like skin disease from the chronic loss of epidermal caspase-8. *Proceedings of the National Academy of Sciences*.

[2] Thestrup-Pedersen K. (2000). Clinical aspects of atopic dermatitis. *Clinical and Experimental Dermatology*.

[3] Chopra R., Vakharia P. P., Sacotte R., Silverberg J. I. (2017). Efficacy of bleach baths in reducing severity of atopic dermatitis: a systematic review and meta-analysis. *Annals of Allergy, Asthma & Immunology*.

[4] Abramovits W., Perlmutter A. (2006). Steroids versus other immune modulators in the management of allergic dermatoses. *Current Opinion in Allergy and Clinical Immunology*.

[5] Sidbury R., Hanifin J. M. (2000). Systemic therapy of atopic dermatitis. *Clinical and Experimental Dermatology*.

[6] Harper J. I., Berth-Jones J., Camp R. D. (2001). Cyclosporin for atopic dermatitis in children. *Dermatology*.

[7] Bajgai J., Fadriquela A., Ara J. (2017). Balneotherapeutic effects of high mineral spring water on the atopic dermatitis-like inflammation in hairless mice via immunomodulation and redox balance. *BMC Complementary and Alternative Medicine*.

[8] Proksch E., Nissen H. P., Bremgartner M., Urquhart C. (2005). Bathing in a magnesium-rich Dead Sea salt solution improves skin barrier function, enhances skin hydration, and reduces inflammation in atopic dry skin. *International Journal of Dermatology*.

[9] Portales P., Aries M. F., Licu D. (2001). Immunomodulation induced by Avene spring water on Th1- and Th2-dependent cytokine production in healthy subjects and atopic dermatitis patients. *Skin Pharmacology and Physiology*.

[10] Stein S., Russak A., Sivan O. (2016). Saline groundwater from coastal aquifers as a source for desalination. *Environmental Science & Technology*.

[11] Kim C. G., Lee J. E., Jeong D. G. (2017). Bathing effects of east saline groundwater concentrates on allergic (atopic) dermatitis-like skin lesions induced by 2,4-dinitrochlorobenzene in hairless mice. *Experimental and Therapeutic Medicine*.

[12] Kim C. G., Kang M., Lee Y. H. (2015). Bathing effects of various seawaters on allergic (atopic) dermatitis-like skin lesions induced by 2,4-dinitrochlorobenzene in hairless mice. *Evidence-Based Complementary and Alternative Medicine*.

[13] Hanifin J. M. (1999). Diagnostic criteria for atopic dermatitis: consider the context. *Archives of Dermatology*.

[14] (1993). Severity scoring of atopic dermatitis: the SCORAD index. *Dermatology*.

[15] Kezic S., Novak N., Jakasa I. (2014). Skin barrier in atopic dermatitis. *Frontiers in Bioscience*.

[16] Elias P. M., Steinhoff M. (2008). “Outside-to-inside” (and now back to “outside”) pathogenic mechanisms in atopic dermatitis. *Journal of Investigative Dermatology*.

[17] Maarouf M., Hendricks A. J., Shi V. Y. (2019). Bathing additives for atopic dermatitis – a systemic review. *Dermatitis*.

[18] Kim H. K., Chang H. K., Baek S. Y. (2012). Treatment of atopic dermatitis associated with *Malassezia sympodialis* by green tea extracts bath therapy: a pilot study. *Mycobiology*.

[19] Lucchetta M. C., Monaco G., Valenzi V. I. (2007). The historical-scientific foundations of thalassotherapy: state of the art. *Clinica Terapeutica*.

[20] Matz H., Orion E., Wolf R. (2003). Balneotherapy in dermatology. *Dermatologic Therapy*.

[21] Boisseau A. M., Donatien P., Surleve-Bazeille J. E. (1992). Production of epidermal sheets in a serum free culture system: a further appraisal of the role of extracellular calcium. *Journal of Dermatological Science*.

[22] Grangsjo A., Pihl-Lundin I., Lindberg M., Roomans G. M. (2003). X-ray microanalysis of cultured keratinocytes: methodological aspects and effects of the irritant sodium lauryl sulphate on elemental composition. *Journal of Microscopy*.

[23] Ludwig P., Petrich K., Schewe T., Diezel W. (1995). Inhibition of eicosanoid formation in human polymorphonuclear leukocytes by high concentrations of magnesium ions. *Biological Chemistry Hoppe-Seyler*.

[24] Greiner J., Diezel W. (1990). Inflammation-inhibiting effect of magnesium ions in contact eczema reactions. *Hautarzt*.

[25] Bak J. P., Kim Y. M., Son J., Kim C. J., Kim E. H. (2012). Application of concentrated deep sea water inhibits the development of atopic dermatitis-like skin lesions in NC/Nga mice. *BMC Complementary and Alternative Medicine*.

